# On the economic burden of alopecia areata in Romania

**DOI:** 10.3389/frhs.2026.1943557

**Published:** 2026-10-21

**Authors:** Mihaela Olaru, Alin Laurentiu Tatu, Rodica Olteanu, Rozalina Lapadatu, Cristian Vladescu, Stefan Strilciuc, Alin Codrut Nicolescu

**Affiliations:** 1 Department of Dermatology, “Saint Parascheva” Infectious Disease Clinical Hospital, Galați, Romania; 2 Department of Clinical Medical, Faculty of Medicine and Pharmacy, “Dunărea de Jos” University of Galați, Galați, Romania; 3 Multidisciplinary Integrated Centre of Dermatological Interface Research (MICDIR), “Dunărea de Jos” University of Galați, Galați, Romania; 4 Dermatology Department, Colentina Clinical Hospital, Bucharest, Romania; 5 Faculty of Medicine, Titu Maiorescu University, Bucharest, Romania; 6 Asociația Pacienților cu Afecțiuni Autoimune (APAA), Bucharest, Romania; 7 National Institute for Management of Health Services, Bucharest, Romania; 8 RoNeuro Institute for Neurological Research and Diagnostic, Cluj-Napoca, Romania; 9 Research Center for Functional Genomics, Biomedicine and Translational Medicine, Iuliu Hațieganu University of Medicine and Pharmacy, Cluj-Napoca, Romania; 10 Carol Davila University of Medicine and Pharmacy, Bucharest, Romania; 11 EgoClinic - Institute of Dermatology, Bucharest, Romania

**Keywords:** alopecia areata, autoimmune, cost of illness, economic burden, healthcare

## Abstract

**Introduction:**

Alopecia areata (AA) is an immune-mediated disorder that substantially impairs quality of life, social participation, and work productivity while generating considerable healthcare costs. Data regarding its economic burden in Romania are currently lacking.

**Methods:**

We conducted a societal-perspective cost-of-illness study for the reference year 2022, combining a cross-sectional online survey of patients and caregivers (41 adult patients and 7 caregiver proxies, of whom 4 reported for pediatric patients) with four national secondary data sources: alopecia areata-specific epidemiological estimates from the Global Burden of Disease (GBD) 2021 study, pharmaceutical market data (IQVIA), hospitalization cost and discharge statistics, and national labor statistics. Survey-derived unit costs were extrapolated to the national level using both prevalence- and incidence-based approaches, combining bottom-up (treatment, medical services, associated expenses), top-down (hospitalizations), and hybrid (productivity losses) costing. Health-related quality of life was assessed with the EQ-5D-5L.

**Results:**

The estimated total cost of alopecia areata in Romania in 2022 was EUR 97.95 million for adults and EUR 3.35 million for pediatric patients under the prevalence-based approach, and EUR 170.56 million and EUR 6.37 million, respectively, under the incidence-based approach. In adults, productivity losses (45.2–51.2%) and associated out-of-pocket expenses (28.5–32.2%) were the largest contributors, followed by medical services and treatment. Among pediatric patients, associated expenses, caregiver productivity losses, and treatment were the main cost drivers. Average annual cost per adult patient was EUR 2,608.6, and was higher for mild (EUR 3,248.3) than for severe disease (EUR 1,999.4). Problems with anxiety/depression were the most frequently affected EQ-5D-5L dimension (77.8% of adult respondents; 6 of 7 caregiver proxies).

**Discussion:**

This is the first study to estimate the economic burden of alopecia areata in Romania. Although the survey sample was small and self-selected, the combination of patient-reported unit costs with national epidemiological and administrative data allowed national burden estimates under transparent assumptions. The findings highlight the substantial societal cost of alopecia areata — driven primarily by productivity losses and out-of-pocket spending rather than by reimbursed care — and support its consideration in healthcare resource allocation and service planning. Larger, representative studies are warranted to validate these estimates.

## 1 Introduction

Alopecia areata (AA) affects patients of all ages, sexes, and ethnicities, with observed variations among group categories (1). For example, in terms of sex differences, some studies point out that men are diagnosed earlier by 4.7 years than women, and 11.2% more men exhibit the first signs at the beard level as opposed to the scalp, while other studies have shown contrary results in terms of sex predominance (2, 3).

The current burden of alopecia areata, expressed through the metrics of the Global Burden of Disease (GBD) study, has remained approximately constant globally. A comprehensive analysis of the epidemiological trends of alopecia areata using global estimates showed slight decreases in global age-standardized incidence rates (ASIRs) compared to 1990 for both men (287.26 vs. 279.18) and women (549.03 vs. 531.68) (4). Another study highlighted a difference of 0.08% between age-standardized prevalence rates in adults and minors (0.18% in adults and 0.10% in pediatric patients), and reported that patients aged 21–40 were the most affected, followed by those aged 40–49 (5). Regarding ethnicity, the highest and lowest prevalence estimates were reported among Asian patients (414/100,000) and White patients (168/100,000), respectively (6). Regarding the geographical distribution of disease burden, countries with high and high-middle sociodemographic index (SDI) were the most affected (ASIRs of 518.91 and 408.62, respectively). In Eastern Europe, ASIRs were similar in 1990 (389.49) and 2019 (387.84) (4).

Beyond epidemiology, AA affects patients and their caregivers on multiple fronts. Shi et al. showed, in a secondary analysis of National Alopecia Areata Registry data, that poor health-related quality of life — identified in 43% of the included patients — was associated with age under 50 years, female sex, greater extent of hair loss, and job- or family-related stress (7). A hospital-based multicenter study conducted in Korea reported that quality of life worsened in family members and pediatric patients as the severity of the disease increased; in the same study, once the disease progressed, the annual cost of treatment exceeded USD 1,000 for most patients (8). Patients affected by this pathology, both adults and children, were more often diagnosed with anxiety and/or depression, or their symptoms, than controls defined as healthy adults (9).

The economic consequences are equally well documented. A retrospective claims-based study of adolescent patients with AA in the United States reported an average total all-cause healthcare cost of USD 9,397 per patient over 12 months of follow-up — versus USD 2,267 among matched controls — of which USD 7,480 represented payer-borne medical costs (10). For the adult patient group (characterized by an average age of 40.3 years and a predominantly female composition — 61%), another US-based claims study reported 12-month all-cause healthcare costs of USD 11,241.21, of which USD 419.12 were attributable to alopecia areata (11). European evidence is consistent: a real-world analysis conducted in Italy estimated the burden attributable to health services at EUR 1,715 per AA patient (12), while a real-world retrospective study conducted in Spain (the PETALO study) estimated mean healthcare costs of EUR 821.2 per patient in the first year after diagnosis (13).

Our study is the first in Romania to examine healthcare utilization, economic burden, and quality of life in patients diagnosed with alopecia areata across age groups. Responses for patients over 18 years were collected from the patients themselves, and for patients under 18 years from their caregivers, who acted as proxies in the data collection process. The primary objective was to estimate the national direct and indirect costs of alopecia areata for adult and pediatric patients in 2022, under both prevalence- and incidence-based approaches; the secondary objectives were to describe treatment patterns, healthcare service utilization, and health-related quality of life in the surveyed sample. By addressing this gap, we aim to offer a policy tool for patients and stakeholders alike, as cost-of-illness studies incorporating quality-of-life instruments are central to policymaking and advocacy.

## 2 Materials and methods

### 2.1 Study design and analytical framework

This cost-of-illness (CoI) study quantified the medical and non-medical costs associated with alopecia areata in Romania over 12 months from a societal perspective, considering direct medical, direct non-medical, and indirect cost components. The reference year was 2022. The study combined one prospective primary data source — a cross-sectional online survey of adult patients and of caregivers of patients with AA (Section 2.2) — with four retrospective secondary data sources: AA-specific epidemiological estimates from the GBD 2021 study, pharmaceutical market data from IQVIA, hospitalization cost estimates from a financial controlling firm together with national hospital discharge statistics, and national labor statistics (Section 2.3).

The analytical sequence was as follows. First, survey responses were used to derive the distribution of disease severity, the shares of patients and caregivers working full-time, self-reported days of absence from work, and mean annual per-patient costs for treatment, medical services, and associated (out-of-pocket, non-medical) expenses, separately for adults and pediatric patients and for mild and severe disease. Second, national numbers of prevalent and incident cases were derived from GBD 2021 estimates and partitioned by severity using the survey-derived proportions. Third, unit costs were combined with the extrapolated case numbers using bottom-up costing for treatment, medical services, and associated expenses; top-down costing for hospitalizations; and a hybrid approach for productivity losses (Section 2.4). All cost components, their input values, and their data sources are summarized in Table 1. Health-related quality of life was assessed with the EQ-5D-5L within the survey (Section 2.5).

**Table 1:** Overview of cost components, inputs, data sources, and costing approaches.

Cost component	Costing approach	Input(s)	Input value	Source
Treatment (direct medical)	Bottom-up	Mean annual per-patient treatment cost	Adults: EUR 344.9 (L1), EUR 281.3 (L2); pediatric: EUR 103.4 (L1), EUR 323.4 (L2)	Survey (imputed with mice where missing)
Treatment — scenario 2	Bottom-up	Average cost per AA prescription	52 RON (EUR 10.54)	IQVIA
Medical services (direct medical)	Bottom-up	Mean annual per-patient cost of medical services	Adults: EUR 391.0 (L1), EUR 351.2 (L2); pediatric: not collected	Survey (imputed with mice where missing)
Hospitalizations (direct medical)	Top-down	Number of AA hospitalizations (main diagnosis L63.9); cost per inpatient day; average length of stay	72 discharges; adults 1,295 RON/day×6.03 days; children 1,321 RON/day×3.85 days	SNSPMN; Hospital Consulting
Associated expenses (direct non-medical)	Bottom-up	Mean annual per-patient out-of-pocket spending on transportation, accommodation, and other AA-related non-medical items	Adults: EUR 700.0 (L1), EUR 784.4 (L2); pediatric: EUR 378.6 (L1), EUR 507.0 (L2)	Survey (imputed with mice where missing)
Productivity losses (indirect)	Hybrid	Self-reported days absent from work (patients; caregivers of pediatric patients); mean gross wage; working days per year; number of full-time workers among prevalent/incident cases	Wage 6,126 RON/month; 251 working days; worker numbers from GBD-based extrapolation and survey employment information	Survey; INSSE; GBD 2021
National case numbers (multipliers)	—	Crude prevalent and incident AA cases, <20 and 20 + years; severity split	Prevalence: 3,792.18 (<20), 37,548.63 (20+); incidence: 6,964.62 (<20), 65,413.8 (20+); severity proportions from survey	GBD 2021; survey

L1 — mild disease (survey severity S0–S2); L2 — severe disease (S3–S5). All costs converted at 1 EUR = 4.9313 RON.

### 2.2 Prospective data source: patient and caregiver survey

We collected primary data through a cross-sectional online questionnaire (data collection: 25 April 2023 to 18 October 2023) built with the SurveyPlanet platform (14) and disseminated on Facebook through targeted advertisements by the Association of Patients with Autoimmune Affections (APAA). Sampling was by convenience, with a target population composed of patients over 18 years old and caregivers of patients with alopecia areata of any age. The questionnaire was fielded as a joint instrument covering atopic dermatitis and alopecia areata; only responses concerning alopecia areata are analyzed in this article. The survey instrument (English and Romanian versions) is provided in Supplementary Material S1; the EQ-5D-5L, administered as part of the survey under license from the EuroQol Research Foundation, is not reproduced there.

The questionnaire was developed based on a literature review of cost-of-illness and health-service-utilization studies and was refined with input from clinicians and the patients’ association to account for the national patient pathway for alopecia areata. The instrument covered three groups of items. The first group captured sociodemographic characteristics (age, gender, settlement type, education, employment, and health insurance status), self-reported disease severity — expressed as the extent of hair loss on six levels: S0 (0% hair loss), S1 (1%–24%), S2 (25%–49%), S3 (50%–74%), S4 (75%–99%), and S5 (100%) — time since diagnosis, and the presence of other health conditions (chronic urticaria, allergic rhinitis, non-allergic rhinitis, food allergies, eosinophilic esophagitis, contact dermatitis, and other autoimmune or inflammatory diseases). The second group covered resource use and spending: the type of each treatment used (topical, oral, injectable, or other), with its dose, frequency of use, and place of administration; utilization of medical services over the preceding 12 months (general practitioner and specialist visits, day and inpatient admissions, laboratory and imaging services); expenses for treatment, medical services, and associated expenses (transportation and accommodation); and days of absence from work for patients and caregivers. Treatment expenses were collected as average monthly amounts and medical-service expenses per visit, in each case distinguishing out-of-pocket payments, payments through voluntary private insurance, and other expenses (with free-text specification); amounts were annualized for the cost analysis. The third group consisted of the EQ-5D-5L instrument (Section 2.5).

The survey yielded 49 responses. One response was excluded because disease severity could not be established, leaving a final analytic sample of 48: 41 adult patients responding for themselves and 7 caregivers responding as proxies — 4 for pediatric patients (aged under 18) and 3 for adult patients. The costing analyses used the 41 self-reporting adult patients for the adult cost model and the 4 caregiver-reported pediatric patients for the pediatric cost model; caregiver responses regarding their own characteristics and absenteeism informed the caregiver productivity component. Descriptive results are reported for all 48 analyzed responses.

### 2.3 Retrospective data sources

#### 2.3.1 Epidemiological estimates: the global burden of disease 2021 study

Numbers of prevalent and incident cases of alopecia areata in Romania were retrieved from the GBD 2021 study — the latest available estimates at the time of analysis — for the year 2021, both sexes combined (15, 16). These are cause-specific estimates for alopecia areata (a distinct cause within the GBD “skin and subcutaneous diseases” category), not estimates for the broader disease category. For the primary (prevalence-based) analysis we used crude prevalent case counts (<20 years: 3,792.18; 20 + years: 37,548.63); for the alternative (incidence-based) analysis we used crude incident case counts (<20 years: 6,964.62; 20 + years: 65,413.8).

We used the two GBD age bands (<20 and 20 + years) because the age bands collected in our survey differed from the GBD groupings; the <20/20 + split covered all surveyed age groups with a single boundary. One surveyed patient aged 18–25 years was assigned to the 20 + band, as this band covers the greater part of that age interval.

GBD 2021 also describes the severity distribution of alopecia areata through two health states: “Disfigurement, level 1” (lay description: “has a slight, visible physical deformity that others notice, which causes some worry and discomfort”) and “Disfigurement, level 2” (“has a visible physical deformity that causes others to stare and comment; as a result, the person is worried and has trouble sleeping and concentrating”) (17). Based on expert opinion and the correspondence between these descriptions and the extent of hair loss, survey severity levels S0–S2 were mapped to L1 (mild) and S3–S5 to L2 (severe). All cost analyses are reported at the L1/L2 level.

#### 2.3.2 Pharmaceutical market data: IQVIA

IQVIA provided the average cost per alopecia areata-related prescription in Romania for the reference year, based on data collected from 3,700 pharmacies: 52 RON (EUR 10.54), including partially and fully reimbursed prescriptions. The estimate excludes so-called “white prescriptions” — the prescription forms used in Romania for medicines that are not reimbursed, i.e., prescriptions paid fully out of pocket, including over-the-counter medicines. This value was used in a scenario analysis as an alternative, conservative estimate of annual treatment cost (Section 2.4.4).

#### 2.3.3 Hospitalization costs: hospital consulting

Hospital Consulting Romania, a financial controlling firm working with Romanian hospitals, provided the average cost per inpatient hospitalization day for patients with alopecia areata in 2022 — 1,295 RON (EUR 262.6) for adults and 1,321 RON (EUR 267.9) for children — and the average length of stay (6.03 days for adults and 3.85 days for children). These estimates are based on yearly updated data from different hospitals, selected on the basis of coverage and profile. Combining the two yields an average cost per hospitalization episode of EUR 1,583.5 for adults and EUR 1,031.3 for children.

#### 2.3.4 Hospital discharge statistics: SNSPMN

The number of alopecia areata-related hospitalizations was retrieved from the National Institute for Health Services Management (SNSPMN) hospital discharge database (18). In 2022 there were 72 hospitalizations with a main discharge diagnosis of “L63.9 — alopecia areata, unspecified” (ICD-10), covering both adult and pediatric patients.

#### 2.3.5 Labor statistics: INSSE

From the National Institute of Statistics (INSSE) we used the mean gross monthly salary for 2022 — 6,126 RON (EUR 1,242) (19) — and the number of working days in 2022 (251) (20), yielding an average cost per working day of 292.9 RON (EUR 59.4). These values were used to monetize absenteeism.

### 2.4 Cost estimation

#### 2.4.1 Cost components and definitions

Costs were structured as: (i) direct medical costs — treatment, medical services (outpatient visits, day admissions, laboratory services), and inpatient hospitalizations; (ii) direct non-medical costs — “associated expenses”, defined as patient- or caregiver-reported out-of-pocket spending on transportation to and from healthcare providers, accommodation occasioned by seeking care, and other alopecia areata-related non-medical items; and (iii) indirect costs — productivity losses due to absenteeism from work of adult patients and of caregivers of pediatric patients, valued with the human capital approach using the national average wage. Presenteeism was not measured. Table 1 links every cost component to its inputs, input values, data sources, and costing approach.

#### 2.4.2 National extrapolation

Based on the surveyed patients (*n* = 41 adult; *n* = 4 pediatric), we calculated the proportion of cases at each severity level and applied these proportions to the GBD-derived numbers of prevalent and incident cases in each age band (<20 and 20 + years) to obtain the national number of mild (L1) and severe (L2) cases. The survey-derived information on full-time employment of patients and, for pediatric patients, of their caregivers was then used to estimate the national number of full-time workers affected, which enters the productivity component. These steps were performed separately under the prevalence-based and the incidence-based approach.

For hospitalizations, the SNSPMN total (72 discharges, adults and children combined) was partitioned across age bands proportionally to the GBD prevalence of each age group, and within age bands across severity levels using the survey-derived severity proportions. The resulting numbers were multiplied by the average cost per hospitalization episode (Section 2.3.3).

#### 2.4.3 Productivity losses

Days absent from work were self-reported for the preceding 12 months by adult patients and by caregivers of pediatric patients. We excluded absence days reported for patients aged over 65 years (considered retired), for patients and caregivers under 65 who declared themselves retired due to illness, and reported values exceeding the number of working days in a year (*n* = 4 responses). Absence days were valued at the average daily wage and combined with the extrapolated numbers of full-time workers (hybrid approach).

#### 2.4.4 Imputation, scenario analysis, and conversion

Missing cost data (for adult patients, across cost variables; for pediatric patients, associated expenses only) were imputed using the “mice” R package (multivariate imputation by chained equations) (21). As a scenario analysis, the survey-derived annual treatment cost was replaced with the IQVIA average cost per prescription (Section 2.3.2), providing a conservative lower bound for the treatment component. All costs were converted into EUR at the European Central Bank average exchange rate for 2022 (1 EUR = 4.9313 RON) (22). We report total costs, cost shares by component, and mean cost per patient by severity level (L1/L2) and age group (<20/20 + years), for both epidemiological approaches and both treatment-cost scenarios.

### 2.5 Health-related quality of life

Health-related quality of life was measured within the survey using the EQ-5D-5L. This instrument comprises five descriptive dimensions (mobility, self-care, usual activities, pain/discomfort, and anxiety/depression), each measured on five levels (no, mild, moderate, severe, and extreme problems), and the EQ-VAS, a visual analogue scale of self-assessed health ranging from 0 (worst) to 100 (best imaginable health) (23). The EQ-5D-5L has been widely used across study designs, including population health surveys, observational studies, and clinical trials (24–27). We chose a single, generic instrument to limit respondent burden, as the survey already exceeded 20 items; the trade-offs of this choice are examined in the Limitations section. Utilities were generated in R using the “eq5d” package (28) with the Romanian EQ-5D-5L value set (29). As recommended by the EQ-5D-5L user guide, we dichotomized responses into “no problems” (level 1) and “problems” (levels 2–5) (30). For the EQ-VAS, we report the mean, standard deviation, median, and 25th and 75th percentiles by age group. Utilities are reported as ranges stratified by severity and sex, given the small number of observations per stratum.

### 2.6 Statistical reporting and ethics

Descriptive results are reported as crude numbers and proportions, stratified by aggregated severity level (L1/L2) with an overall column, consistent with the groupings used in the economic analyses. Because of the small sample, no inferential statistics were performed, and cells based on very few observations (*n* < 5) are interpreted descriptively only. The Institutional Review Board — Public Health (IRB-PH) of Babeș-Bolyai University approved the research protocol (no. 151122-003). Participation was voluntary and responses were anonymous; completion of the online questionnaire implied informed consent.

## 3 Results

### 3.1 Survey sample and baseline characteristics

*Data source: prospective survey — adult patients (self-report, n* *=* *41) and caregiver proxies (n* *=* *7, reporting on 4 pediatric and 3 adult patients).*

Of the 41 adult respondents, 20 (48.8%) reported mild disease (L1: severity S0–S2) and 21 (51.2%) severe disease (L2: S3–S5). Most adult patients were female, aged between 36 and 55 years, resided in urban areas, had post-secondary or university education, were employed full-time, and had been diagnosed either within the previous 1–3 years or more than 10 years earlier (Table 2). Patients with severe disease were more often female (85.7% vs. 75.0%), less often employed full-time (47.6% vs. 65.0%), more often retired (42.9% vs. 20.0%), and markedly more often diagnosed more than 10 years earlier (47.6% vs. 20.0%) than patients with mild disease. Of the 7 caregiver-reported patients, 6 had mild and 1 had severe disease; 4 were children or adolescents (Table 2).

**Table 2:** Baseline characteristics of surveyed patients: adult patients (self-report) by aggregated severity level, and caregiver-reported patients.

Characteristic	Adults L1 (*n* = 20)	Adults L2 (*n* = 21)	Adults total (*N* = 41)	Caregiver-reported (*N* = 7)
Sex				
Male	5 (25.0%)	3 (14.3%)	8 (19.5%)	4 (57.1%)
Female	15 (75.0%)	18 (85.7%)	33 (80.5%)	3 (42.9%)
Age group (patient)				
1–10 years	—	—	—	1 (14.3%)
11–17 years	—	—	—	3 (42.9%)
18–25 years	1 (5.0%)	0	1 (2.4%)	—
26–35 years	4 (20.0%)	1 (4.8%)	5 (12.2%)	1 (14.3%)
36–45 years	6 (30.0%)	7 (33.3%)	13 (31.7%)	1 (14.3%)
46–55 years	6 (30.0%)	5 (23.8%)	11 (26.8%)	—
56–65 years	1 (5.0%)	4 (19.0%)	5 (12.2%)	1 (14.3%)
66–75 years	2 (10.0%)	4 (19.0%)	6 (14.6%)	—
Settlement type				
Urban	12 (60.0%)	10 (47.6%)	22 (53.7%)	6 (85.7%)
Rural	8 (40.0%)	11 (52.4%)	19 (46.3%)	1 (14.3%)
Education level (patient)				
Kindergarten/nursery	—	—	—	1 (14.3%)
Primary school	2 (10.0%)	2 (9.5%)	4 (9.8%)	2 (28.6%)
Gymnasium	1 (5.0%)	2 (9.5%)	3 (7.3%)	1 (14.3%)
High school	3 (15.0%)	3 (14.3%)	6 (14.6%)	1 (14.3%)
Post-secondary school	6 (30.0%)	6 (28.6%)	12 (29.3%)	—
University	8 (40.0%)	8 (38.1%)	16 (39.0%)	2 (28.6%)
Employment status ^a^				
Employed full-time	13 (65.0%)	10 (47.6%)	23 (56.1%)	3 (42.9%)
Employed part-time	1 (5.0%)	1 (4.8%)	2 (4.9%)	—
Unemployed	1 (5.0%)	1 (4.8%)	2 (4.9%)	—
Student	—	—	—	1 (14.3%)
Retired (age limit)	3 (15.0%)	7 (33.3%)	10 (24.4%)	2 (28.6%)
Retired (due to sickness)	1 (5.0%)	2 (9.5%)	3 (7.3%)	1 (14.3%)
Maternity leave	1 (5.0%)	0	1 (2.4%)	—
Time since diagnosis				
Under 1 year	1 (5.0%)	1 (4.8%)	2 (4.9%)	1 (14.3%)
1–3 years	8 (40.0%)	6 (28.6%)	14 (34.1%)	4 (57.1%)
4–6 years	2 (10.0%)	1 (4.8%)	3 (7.3%)	2 (28.6%)
7–9 years	5 (25.0%)	3 (14.3%)	8 (19.5%)	—
Over 10 years	4 (20.0%)	10 (47.6%)	14 (34.1%)	—

L1 — mild disease (severity S0–S2); L2 — severe disease (S3–S5). Percentages are column percentages.

a For caregiver-reported patients, employment status refers to the caregiver. In the originally submitted version, the four patients aged 66–75 years with severity S3 were misprinted in the S2 column; this has been corrected.

### 3.2 Comorbidities

*Data source: prospective survey.*

Among adult respondents, the most frequently reported co-occurring conditions were autoimmune thyroiditis (*n* = 13), atopic dermatitis (*n* = 8), psoriasis (*n* = 7), systemic lupus erythematosus (*n* = 4), and anemia (*n* = 3); 10 respondents reported none of the listed conditions. Caregivers reported atopic dermatitis (*n* = 1) and anemia (*n* = 1) in the patients they cared for, while most (*n* = 5) reported none of the listed conditions.

### 3.3 Treatment patterns

*Data source: prospective survey.*

Among adult respondents, 24 used ointments, 17 oral treatments, 8 injections, and 7 other forms (shampoo/shower gel, *n* = 2; infusions, *n* = 1). Most applied or took their treatment once per day (*n* = 15) or multiple times per day (*n* = 10); 6 used it once per week, 4 only when needed, and 6 could not specify the frequency. All 7 caregivers reported that the patients they cared for used ointments; one also reported an oral treatment, one an injectable treatment, and one an anti-hair-fall serum. Most caregiver-reported patients used their treatment once or multiple times per day (*n* = 6); one used it when needed.

### 3.4 Healthcare service utilization

*Data source: prospective survey; 12-month recall.*

Fifteen adult patients reported visiting their general practitioner for alopecia areata during the preceding 12 months (reported frequencies between 0 and 10 visits, most commonly 3, 6, or 9), and one further patient reported GP visits for other reasons. Twenty-three adult patients reported visiting a specialist physician, with frequencies in a similar range. Seven adult patients reported day (outpatient) admissions (2 to 12 admissions per patient over the year) and six reported inpatient admissions (5 to 9 admissions per patient). Eleven adult patients used medical laboratory services in connection with alopecia areata; the reported number of utilizations was mostly between 1 and 8, with two respondents each reporting 98 utilizations. Among caregivers, reported specialist visit frequencies ranged from 2 to 13; six of seven caregivers reported no day admissions for the patient in their care (one reported a 2-day admission), and similar results were reported for inpatient admissions. Five caregivers reported use of laboratory services, with 2 to 12 utilizations over the year.

Given the small sample, these utilization figures — particularly values reported by single respondents — should be read as descriptive observations rather than population estimates.

### 3.5 Work absenteeism

*Data source: prospective survey; 12-month recall.*

Among adult patients reporting absence from work due to alopecia areata, the number of days ranged from 1 (*n* = 2) to 174 (*n* = 1); the remaining reported values were 3, 5, 6, 10, 12, 13, 16, and 20 days. Caregivers reported that the patients they cared for missed 3, 13, 15, 19, and 300 days of work or education, and reported 2, 3, 20, and 50 days of absence from their own work due to the patients’ alopecia areata. Values exceeding the annual number of working days were excluded from the costing analyses (Section 2.4.3).

### 3.6 National extrapolation

*Data sources: GBD 2021, survey-derived severity proportions and employment information, SNSPMN.*

Applying the survey-derived severity proportions to the GBD 2021 case counts yielded, under the prevalence-based approach, 18,317 mild (L1) and 19,232 severe (L2) cases among people aged 20 + years, and 2,845 L1 and 948 L2 cases among those aged under 20. Under the incidence-based approach, the corresponding figures were 31,909 L1 and 33,505 L2 cases (20 + years) and 5,224 L1 and 1,741 L2 cases (<20 years) (Table 3).

**Table 3:** Extrapolated national case numbers, full-time workers, and hospitalizations, by age band and severity level.

Quantity	Age band	Prevalence-based	Incidence-based
Cases, L1	<20 years	2,845	5,224
Cases, L2	<20 years	948	1,741
Cases, L1	20 + years	18,317	31,909
Cases, L2	20 + years	19,232	33,505
Full-time workers (patients), L1	20 + years	12,211	21,273
Full-time workers (patients), L2	20 + years	6,411	11,168
Full-time workers (caregivers), L1 + L2	<20 years	1,897	3,823
Hospitalizations, L1/L2	<20 years	5/2	5/2
Hospitalizations, L1/L2	20 + years	32/33	32/33

L1 — mild disease; L2 — severe disease. Sources: GBD 2021 (case counts), survey (severity proportions and employment information), SNSPMN (hospitalizations).

Based on the survey-derived employment information, an estimated 12,211 L1 and 6,411 L2 adult patients were full-time workers under the prevalence-based approach (21,273 and 11,168, respectively, under the incidence-based approach). The estimated number of full-time-working caregivers of pediatric patients was 1,897 (prevalence-based) and 3,823 (incidence-based). Partitioning the 72 alopecia areata hospitalizations recorded in 2022 yielded 7 hospitalizations among patients under 20 years (5 L1, 2 L2) and 65 among patients aged 20+ (32 L1, 33 L2); these numbers were identical under both epidemiological approaches (Table 3).

### 3.7 Economic burden

*Data sources: survey-derived unit costs, IQVIA, Hospital Consulting, SNSPMN, INSSE, GBD 2021 (* Table 1 *).*

#### 3.7.1 Adult patients

The total cost of alopecia areata for adult patients in Romania in 2022 was EUR 97,950,652.6 under the prevalence-based approach (EUR 59,498,429.4 for mild and EUR 38,452,223.1 for severe cases) and EUR 170,562,808.4 under the incidence-based approach (Table 4). Productivity losses were the largest component (45.2% of the total under both approaches), followed by associated expenses (28.5%), medical services (14.2%), treatment (12.0%), and hospitalizations (<0.1%).

**Table 4:** Total annual costs for adult patients (20 + years), by component, severity level, and epidemiological approach (EUR, 2022).

Cost component	Prevalence-based, L1	Prevalence-based, L2	Prevalence-based, total (%)	Incidence-based, L1	Incidence-based, L2	Incidence-based, total (%)
Treatment	6,318,256.2	5,409,280.3	11,727,536.5 (11.97%)	11,006,673.5	9,423,770.0	20,430,443.5 (11.98%)
Medical services	7,161,433.3	6,754,775.4	13,916,208.7 (14.21%)	12,475,524.1	11,767,822.0	24,243,346.1 (14.21%)
Hospitalizations	50,672.9	52,256.4	102,929.3 (0.11%)	50,672.9	52,256.4	102,929.3 (0.06%)
Associated expenses	12,814,805.0	15,085,145.0	27,899,950.0 (28.48%)	22,323,941.0	26,280,563.0	48,604,504.0 (28.50%)
Productivity losses	33,153,262.0	11,150,766.0	44,304,028.0 (45.23%)	57,756,886.2	19,424,700.0	77,181,586.2 (45.25%)
**Total**	**59,498,429.4**	**38,452,223.1**	**97,950,652.6 (100%)**	**103,613,697.7**	**66,949,111.4**	**170,562,808.4 (100%)**
Treatment — scenario 2 ^a^	193,150.7	202,799.3	395,950.0 (0.46%)	—	—	(0.46%)

L1 — mild disease; L2 — severe disease.

a Scenario 2 replaces the survey-derived treatment cost with the IQVIA average cost per prescription; under this scenario the shares of the remaining components change accordingly (e.g., productivity losses 51.15% under the prevalence-based and 51.17% under the incidence-based approach).

Bold represents totals.

In the scenario replacing survey-reported treatment costs with the IQVIA average cost per prescription, the treatment component fell to EUR 193,150.7 (L1) and EUR 202,799.3 (L2) under the prevalence-based approach, reducing the treatment share of total costs to 0.5% and increasing the relative weight of productivity losses to 51.2% (Table 4).

#### 3.7.2 Pediatric patients

The total cost of alopecia areata for pediatric patients in Romania in 2022 was EUR 3,348,772.7 under the prevalence-based approach and EUR 6,373,828.4 under the incidence-based approach (Table 5). Associated expenses were the largest component (41.8% and 40.4% of the total, respectively), followed by caregiver productivity losses (38.5% and 40.7%) and treatment (19.5% and 18.8%); hospitalizations accounted for less than 0.3%. Medical services costs were not collected for pediatric patients and are therefore not included (Section 2.4.1). In the IQVIA treatment-cost scenario, the pediatric treatment component fell to EUR 30,000.2 (L1) and EUR 9,996.5 (L2) under the prevalence-based approach and to EUR 55,086.5 (L1) and EUR 18,358.7 (L2) under the incidence-based approach.

**Table 5:** Total annual costs for pediatric patients (<20 years), by component, severity level, and epidemiological approach (EUR, 2022).

Cost component	Prevalence-based, L1	Prevalence-based, L2	Prevalence-based, total (%)	Incidence-based, L1	Incidence-based, L2	Incidence-based, total (%)
Treatment	294,232.8	358,914.7	653,147.5 (19.50%)	540,271.3	659,146.1	1,199,417.4 (18.82%)
Hospitalizations	5,156.7	2,062.7	7,219.4 (0.22%)	5,156.7	2,062.7	7,219.4 (0.11%)
Associated expenses	920,198.5	480,603.5	1,400,802.0 (41.83%)	1,689,672.0	882,627.3	2,572,299.3 (40.36%)
Caregiver productivity losses	241,425.8	1,046,178.0	1,287,603.8 (38.45%)	486,542.3	2,108,350.0	2,594,892.3 (40.71%)
**Total**	**1,461,013.8**	**1,887,758.9**	**3,348,772.7 (100%)**	**2,721,642.3**	**3,652,186.1**	**6,373,828.4 (100%)**
Treatment — scenario 2 ^a^	30,000.2	9,996.5	39,996.7 (1.46%)	55,086.5	18,358.7	73,445.2 (1.40%)

L1 — mild disease; L2 — severe disease. Medical services costs were not collected for pediatric patients.

a Scenario 2 replaces the survey-derived treatment cost with the IQVIA average cost per prescription.

Bold values represent totals.

#### 3.7.3 Average cost per patient

The average annual cost per adult patient was EUR 2,608.6 under the prevalence-based approach and EUR 2,607.4 under the incidence-based approach, decreasing to EUR 2,306.8 and EUR 2,305.7 in the IQVIA treatment-cost scenario (Table 6). The average cost was consistently higher for mild (L1: EUR 3,248.3, prevalence-based) than for severe cases (L2: EUR 1,999.4). For pediatric patients, the average annual cost per patient was EUR 882.9 (prevalence-based) and EUR 915.1 (incidence-based); in contrast to adults, the pediatric average was higher for severe (L2: EUR 1,991.3) than for mild cases (L1: EUR 513.5).

**Table 6:** Average annual cost per patient (EUR, 2022), by age group, severity level, epidemiological approach, and treatment-cost scenario.

Group	Prevalence-based	Incidence-based	Prevalence-based, scenario 2	Incidence-based, scenario 2
Adults (20 + years)				
All	2,608.6	2,607.4	2,306.8	2,305.7
L1 (mild)	3,248.3	3,247.2	2,913.9	2,912.8
L2 (severe)	1,999.4	1,998.2	1,728.7	1,727.5
Pediatric (<20 years)				
All	882.9	915.1	721.2	753.5
L1 (mild)	513.5	521.0	420.7	428.1
L2 (severe)	1,991.3	2,097.8	1,623.3	1,729.7

Scenario 2 replaces the survey-derived treatment cost with the IQVIA average cost per prescription.

### 3.8 Health-related quality of life

*Data source: prospective survey (EQ-5D-5L completed by 27 of 41 adult respondents and by all 7 caregiver proxies; three dimensions were missing for 2 proxies).*

#### 3.8.1 EQ-5D-5L dimensions

Anxiety/depression was the most affected dimension: 21 of 27 adult respondents (77.8%) and 6 of 7 caregiver proxies reported problems (Table 7). Pain/discomfort followed (19/27 adults, 70.4%; 5/7 proxies). Problems with usual activities (9/27) and self-care (4/27) were less frequent among adults, and no adult respondent reported mobility problems; among proxies, 2 of 5 reported problems on each of the mobility, self-care, and usual-activities dimensions.

**Table 7:** Frequencies of respondents reporting problems (EQ-5D-5L level ≥ 2) on each dimension.

EQ-5D-5L dimension	Adult patients (*n* = 27), problems *n* (%)	Caregiver-reported patients, problems n/n available
Mobility	0 (0%)	2/5
Self-care	4 (14.8%)	2/5
Usual activities	9 (33.3%)	2/5
Pain/discomfort	19 (70.4%)	5/7
Anxiety/depression	21 (77.8%)	6/7

Proxy responses on the mobility, self-care, and usual-activities dimensions were available for 5 of 7 caregiver-reported patients.

#### 3.8.2 EQ-VAS

The overall mean EQ-VAS among adult respondents was 51.0, with age-band means between 45.5 (36–45 years) and 66.0 (56–65 years) (Table 8). Among caregiver-reported patients, EQ-VAS values ranged from 30 to 100, with most bands represented by a single observation.

**Table 8:** EQ-VAS by age band: adult patients and caregiver-reported patients.

Age band	Adult patients, mean (SD) [median; P25–P75]	Caregiver-reported, mean (SD) [median; P25–P75]
1–10 years	—	100 (*n* = 1)
11–17 years	—	45 (35) [60; 32.5–65]
18–25 years	70 (*n* = 1)	—
26–35 years	45.6 (36.1) [50; 10–80]	91 (*n* = 1)
36–45 years	45.5 (29.4) [50; 10–70]	30 (*n* = 1)
46–55 years	59.6 (22.7) [70; 41–75.5]	—
56–65 years	66.0 (16.3) [60; 54–75]	70 (*n* = 1)
66–75 years	48.5 (37.3) [70; 18.25–73.75]	—

#### 3.8.3 Utilities

Among adult respondents, calculated utilities ranged between 0.136 and 1 for females and between 0.083 and 0.738 for males. Stratified by disease severity, utility ranges were 0.711–0.801 for S0, 0.083–1 for S1, 0.701–0.962 for S2, 0.205–0.947 for S3, 0.803–0.941 for S4, and 0.671–0.909 for S5. Among caregiver-reported patients, utilities ranged between 0.734 and 1 for females; the single male patient had a utility of 0.704. By severity, the corresponding values were 0.734 for S1, 0.941–1 for S2, and 0.704 for S3. Given the very small strata, these ranges should be interpreted with caution and are provided for descriptive purposes only.

## 4 Discussion

To our knowledge, this is the first study to estimate the economic burden and self-rated health of patients diagnosed with alopecia areata in Romania, covering both adults and children. The estimated societal cost in 2022 was EUR 97.95 million for adults and EUR 3.35 million for pediatric patients under the prevalence-based approach (approximately EUR 101.3 million in total), rising to EUR 176.9 million in total under the incidence-based approach. For adult patients, productivity losses (45.2–51.2%) and associated out-of-pocket expenses (28.5–32.2%) contributed the largest shares, followed by medical services and treatment, with hospitalizations accounting for a negligible fraction. For pediatric patients, associated expenses, caregiver productivity losses, and treatment were the dominant components. The concentration of the burden in productivity losses and out-of-pocket spending, rather than in reimbursed inpatient care, indicates that most of the cost of alopecia areata in Romania is currently borne by patients, their families, and employers rather than by the health system.

The average annual cost per adult patient (EUR 2,608.6) is of the same order of magnitude as estimates from other settings once differences in scope are considered. In the United States, all-cause 12-month healthcare costs were USD 9,397 among adolescents and USD 11,241 among adults with AA, with AA-attributable payer costs of USD 419 in adults (10, 11); our estimate, by contrast, reflects AA-attributable costs from a societal perspective, most of which fall outside the payer's budget. In Italy, the health-services burden attributable to AA was estimated at EUR 1,715 per patient (12) — comparable to our combined treatment and medical-services average — while in Spain mean first-year healthcare costs were EUR 821.2 per patient (13). Out-of-pocket spending has consistently emerged as a major burden component internationally: in German-speaking countries, patients reported average monthly out-of-pocket costs of EUR 38.4 (± 59.8) for hair-related items and EUR 15.3 (± 32.9) for medicines (31), and a National Alopecia Areata Foundation survey in the United States documented median annual AA-related out-of-pocket costs of USD 1,354 (interquartile range USD 537–3,300), driven by headwear and cosmetic products, co-payments, and medications (32). Our finding that associated expenses represent 28%–42% of the national burden is consistent with this pattern and suggests that the out-of-pocket burden documented in higher-income settings also applies — proportionally — in Romania.

The average annual cost per adult patient was, counterintuitively, higher for mild (L1: EUR 3,248.3) than for severe disease (L2: EUR 1,999.4). Several mechanisms plausibly contribute. First, and most importantly, the composition of the two groups differs with respect to labor-market participation: full-time workers were concentrated in the mild group (extrapolated 12,211 vs. 6,411 full-time workers under the prevalence-based approach), whereas retirement, which excludes patients from the absenteeism component altogether, was twice as frequent in the severe group (42.9% vs. 20.0% in the survey). Because productivity losses are the single largest cost component, a severity group with fewer active workers mechanically accrues lower total and average costs. Second, survey-reported spending on treatment and medical services was somewhat higher among mild patients (EUR 344.9 vs. 281.3 and EUR 391.0 vs. 351.2, respectively), a pattern compatible with more intensive treatment-seeking early in the disease course; patients with long-standing severe disease may instead reduce spending after repeated therapeutic disappointment, and nearly half of the L2 group had been diagnosed more than 10 years earlier. Consistent with this interpretation, health-related quality-of-life impairment (and with it, active care-seeking) is documented across the severity spectrum in alopecia areata, with even limited hair loss producing meaningful burden (33, 34). Third, with 41 respondents providing the unit costs, sampling variability cannot be excluded, and severity was self-reported. Notably, the gradient was reversed in the pediatric population, where severe cases cost nearly four times more than mild ones, driven by treatment and caregiver absenteeism. This reversal suggests that the adult pattern reflects the composition effects above rather than a genuinely lower need in severe disease, and the finding should therefore not be read as evidence that severe alopecia areata is inexpensive; it primarily reflects where the measurable, monetized burden currently falls.

Anxiety/depression was by far the most affected EQ-5D-5L dimension, reported as problematic by 77.8% of adult respondents and 6 of 7 caregiver proxies, and mean EQ-VAS scores (overall 51.0) were low for a dermatological condition — the most consistent signal in our data. This aligns with a systematic review and meta-analysis of anxiety, depression, and quality of life in AA, in which individual studies reported anxiety prevalence between 8% and 19.1% in patients versus controls, separation anxiety (up to 8%) and generalized anxiety disorder (up to 16%) among pediatric patients, and a moderate overall effect of AA on quality of life (9). Population-based studies confirm elevated rates of depressive episodes and anxiety in AA in UK primary care (35), and alopecia areata has been identified as a proximal risk factor for the subsequent development of depression (36). In claims data, depression and anxiety are among the most frequent comorbidities recorded in patients with AA (11). Beyond the patients themselves, caregivers of children with alopecia areata report considerable emotional burden and reduced quality of life (37), and worse quality of life among minor patients corresponds with worse quality of life among their caregivers (8). Taken together with our cost findings, these results argue for treating alopecia areata not as a cosmetic complaint but as a condition with a substantial mental-health footprint: psychological support for patients and family members, and reimbursement pathways for auxiliary services such as counseling — currently reimbursed under restrictive conditions in Romania — are directly relevant to reducing the burden documented here.

The comorbidity profile of our sample (dominated by autoimmune thyroiditis, atopic dermatitis, and psoriasis) is consistent with the autoimmune clustering described in claims-based and registry studies, in which thyroid disorders, atopic dermatitis, and psychiatric conditions are among the most frequent co-diagnoses in patients with AA (11). Reported sex distribution in AA cohorts varies: some studies report female predominance (38, 39), while others report male predominance or no clear difference (2, 3, 5). Our sample showed a marked female predominance (80.5% of adult respondents), and the age distribution, concentrated in the 36–55-year bands, is broadly consistent with cross-sectional survey studies of AA, in which 83% of respondents were female and most were aged 45 years or older (38). Both features, however, are also compatible with the recruitment channel we used, as discussed under Limitations.

Regarding healthcare utilization, general practitioners and specialist physicians provided the most used services, while day and inpatient admissions were rare — mirrored, at the national level, by only 72 AA hospitalizations recorded in 2022. This pattern is consistent with a condition managed almost entirely in ambulatory care, and it implies that access to primary and specialist dermatological services is the operative constraint for patients with alopecia areata. In Romania, unequal geographical distribution of and access to health services has been extensively documented (40, 41), and almost half of our adult respondents (46.3%) resided in rural areas, where the density of dermatological services is lowest. Service planning for alopecia areata should therefore focus on ambulatory dermatology capacity and its territorial distribution rather than on hospital-based care.

Productivity losses were the largest single component of the adult burden under all scenarios (45.2–51.2%). Our study captured only absenteeism valued at the average wage; it did not capture presenteeism — reduced productivity while at work — which the literature suggests is substantial in AA, given the documented association of AA-related emotional symptoms with impaired work productivity and daily activity (42, 43). Patients with AA also face a higher risk of job loss and difficulties in finding employment, partly mediated by the mental-health complications of the disease (35, 38, 43). In our study, the average absenteeism cost was EUR 340.4 (L1) and EUR 434.8 (L2) per adult patient, and EUR 127.3 (L1) to EUR 551.5 (L2) per caregiver of a pediatric patient; the monetary weight patients themselves attach to the disease is illustrated by a US clinic sample in which the median willingness to pay for a permanent cure was USD 500–1,000, corresponding to roughly 10%–20% of monthly income (44). Future Romanian studies should incorporate a validated instrument such as the Work Productivity and Activity Impairment (WPAI) questionnaire (45) to capture both absenteeism and presenteeism.

### 4.1 Strengths

Our study combines a nationally representative epidemiological source — AA-specific estimates from the GBD 2021 study — with national hospital discharge statistics, market-level pharmaceutical data, and patient-reported unit costs, and it quantifies the burden for adults and children alike. It captures associated out-of-pocket costs, which are frequently overlooked when analyses focus only on direct medical or indirect costs, and it presents estimates under both prevalence- and incidence-based approaches with an explicit scenario analysis for the least certain input (treatment costs). All inputs and their sources are reported transparently (Table 1), allowing the estimates to be reproduced or updated as better data become available.

### 4.2 Limitations

The estimates presented here should be interpreted in light of several limitations, most of which stem from the questionnaire-based design.

First, the survey used convenience sampling through a single social media platform (Facebook), with recruitment mediated by a patients’ association. This design carries a substantial risk of selection bias: respondents are more likely to be digitally literate, engaged with patient organizations, and more severely affected or more health-conscious than the average patient, and no response rate can be computed. The marked female predominance (80.5%), the urban and highly educated profile of the sample, and the high proportion of severe cases may all partly reflect this recruitment channel rather than the underlying patient population; the literature on sex distribution in AA is itself mixed (2, 3, 5). Because severity proportions from this sample were used as national multipliers, any such bias propagates to the cost estimates. We partially mitigated this by presenting two epidemiological approaches and a conservative treatment-cost scenario, but representativeness cannot be established. The restriction to one platform also means that patients active on other channels were not reached (46).

Second, the sample was very small: 41 adult patients, and only 4 caregiver-reported pediatric patients supporting the pediatric cost model, with 7 caregivers overall. Survey-derived unit costs and severity proportions are therefore imprecise, several results rest on single observations (reported descriptively only and not treated as findings), and no inferential statistics were attempted. The pediatric estimates in particular should be regarded as exploratory. The low number of respondents may be explained by the comparatively low prevalence of alopecia areata relative to other skin diseases, by undiagnosed cases, and by patients not seeking medical care for mild forms (5, 47).

Third, participants reported healthcare utilization, expenses, and days of absence over the preceding 12 months, exposing all survey-derived inputs to recall bias; annual expenses recalled retrospectively are prone to both under- and over-estimation.

Fourth, apart from the EQ-5D-5L, the questionnaire was developed *ad hoc* for this study; although it was constructed from the cost-of-illness literature and refined with clinician and patient-association input, it was not formally validated. Cost categories may therefore have been understood heterogeneously by respondents — particularly the “associated expenses” category, which we defined for respondents as transportation, accommodation, and other AA-related non-medical spending.

Fifth, several components of the burden were not measured, making our totals conservative: medical services costs for pediatric patients were not collected; specific high-cost items such as wigs were not itemized; presenteeism was not captured; caregiver burden was measured only through absenteeism days, without distinguishing formal from informal caregivers and without capturing paid care or psychological counseling costs (37).

Sixth, the extrapolation relies on GBD 2021 modeled estimates applied to the 2022 reference year, on a severity mapping from self-reported hair-loss extent to the two GBD disfigurement health states based on expert opinion, and on hospitalization unit costs from a private financial-controlling source; missing cost data were imputed (mice). The alternative treatment-cost scenario based on the IQVIA per-prescription average is conservative by construction, as it excludes non-reimbursed (“white prescription”) spending. Each of these steps introduces uncertainty that is not reflected in the point estimates.

Finally, we relied on a single, generic quality-of-life instrument. The EQ-5D-5L was chosen to limit respondent fatigue in an already long survey (48, 49), is validated across populations and study designs, and has a recently published Romanian value set (29); its use also makes our utilities directly usable in future economic evaluations. However, dermatology-specific instruments (DLQI (50), Scalpdex 51) or disease-specific instruments (Alopecia Areata Symptom Impact Scale 52) would likely capture the specific functional and emotional burden of hair loss with greater sensitivity, and instruments quantifying mental-health impact (e.g., the Hospital Anxiety and Depression Scale or the Beck inventories) would allow the psychological burden observed here to be characterized clinically (43). The EQ-5D-5L is also not designed for young children, so proxy-reported utilities for the youngest patients should be interpreted cautiously. Future studies should combine a generic utility instrument with disease-specific and mental-health measures.

### 4.3 Conclusions

Alopecia areata generated an estimated societal cost of EUR 101.3–176.9 million in Romania in 2022, borne predominantly by patients, families, and employers through out-of-pocket spending and lost productivity rather than by the health system through reimbursed care. The disease carries a substantial psychological burden, with anxiety/depression problems reported by the large majority of respondents. These findings provide a first, transparent evidence base for considering alopecia areata in healthcare resource allocation, ambulatory dermatology service planning, and reimbursement decisions in Romania. Larger, representative studies — incorporating validated productivity and disease-specific quality-of-life instruments — are warranted to validate and refine these estimates.

## Data Availability

The original contributions presented in the study are included in the article/Supplementary Material, further inquiries can be directed to the corresponding authors.
