# Supplementary material for: On the economic burden of alopecia areata in Romania
Source: Front Health Serv. 2026 Oct 21;6:1943557. doi: 10.3389/frhs.2026.1943557 (PMC13731240; doi:10.3389/frhs.2026.1943557)
Supplement: Supplementary file 1 [file Table1.docx]

# **Supplementary Material S1**

**Survey instrument — “The economic burden of atopic dermatitis and alopecia areata in Romania” (English and Romanian versions)**

The questionnaire below was administered online (SurveyPlanet) between 25 April 2023 and 18 October 2023, with recruitment through targeted Facebook advertisements placed by the Association of Patients with Autoimmune Affections (APAA). The instrument was designed to cover both atopic dermatitis and alopecia areata; only responses concerning alopecia areata are analyzed in the present article. The EQ-5D-5L, administered as part of the survey under license from the EuroQol Research Foundation, is a proprietary instrument and is not reproduced here. Question sets for treatments and medical services were generated dynamically by the survey platform according to the number of treatments or services reported by the respondent.

Non-eligible participants received the following exit message: “This study is directed toward adults with atopic dermatitis and/or alopecia areata. Thank you for your interest and have a nice day!”

## English version

### Screening

| Item | Response options |
| --- | --- |
| Introduction and screening informed consent | (from attached template) - Consent/Do not consent |
| Are you age 18 or older? | Yes/No |
| I self-identify as suffering from (multiple choice) | Atopic dermatitis; Alopecia areata; None of the above (redirect to questionnaire end) |

### Introduction

| Item | Response options |
| --- | --- |
| Introduction and informed consent | (from attached template) - Consent/Do not consent |

### Demographics

| Item | Response options |
| --- | --- |
| Please select what best describes your situation: |  |
| Gender (single choice) | Female; Male; Other |
| Age | (numeric, integer) |
| Residence (single choice) | Urban; Rural |
| Education (single choice) | No formal education; Primary school; Secondary school; High school; Post-secondary school; University |
| Employment (single choice) | Student; Employed full-time; Employed part-time; Unemployed; Retired; Other |

### Medical Information

| Item | Response options |
| --- | --- |
| Please describe what best describes your situation: |  |
| NHIH insurance status (single choice) | Insured; Uninsured; Co-insured; I don’t know |
| Medical diagnosis of atopic dermatitis (if applicable) | I have a diagnosis from a medical doctor; I don’t have a diagnosis from a medical doctor |
| Medical diagnosis of alopecia areata (if applicable) | I have a diagnosis from a medical doctor; I don’t have a diagnosis from a medical doctor |
| Months since atopic dermatitis diagnosis (if applicable) | (numeric, integer) |
| Months since alopecia areata diagnosis (if applicable) | (numeric, integer) |
| Severity of atopic dermatitis at diagnosis (if applicable) (single choice) | Mild; Moderate; Severe; I don’t know |
| Control of atopic dermatitis symptoms now (4 point Likert) | Not at all under control; Sometimes under control; Most times under control; Always under control |
| Severity of alopecia areata at diagnosis (if applicable) (single choice) | S0 (no hair loss); S1 (1-24% hair loss); S2 (25-49% hair loss); S3 (50-74% hair loss); S4 (75-99% hair loss); S5 (100% hair loss); I don’t know |

### Use Of Treatment

| Item | Response options |
| --- | --- |
| Please describe your current treatment regimen: |  |
| ATOPIC DERMATITIS (list several if applicable) |  |
| Number of treatments used (platform will generate clusters of questions based on number of treatments used) | (numeric, integer) |
| Treatment name | (open ended) |
| Treatment type (single choice) | Topical; Oral; Injectable; Other |
| Treatment dose | (open ended) |
| Frequency of use (single choice) | Once a week; Once a day; Multiple times a day; As needed, during flare-ups; I don’t know |
| Location of administration | At home; Doctor’s office; Hospital; Other |
| Estimated treatment cost (per month average) |  |
| Out-of-pocket (RON) - amount spent | (numeric, integer) |
| Voluntary private insurance (RON) - amount spent | (numeric, integer) |
| Other costs with treatment (RON) - please specify what: | (open ended) |
| Other costs with treatment (RON) - amount spent | (numeric, integer) |
| ALOPECIA AREATA (if applicable) |  |
| Number of treatments used (platform will generate clusters of questions based on number of treatments used) | (numeric, integer) |
| Topical treatment name | (open ended) |
| Treatment type (single choice) | Topical; Oral; Injectable; Other |
| Treatment dose | (open ended) |
| Frequency of use (single choice) | Once a week; Once a day; Multiple times a day; As needed, during flare-ups; I don’t know |
| Location of administration | At home; Doctor’s office; Hospital; Other |
| Estimated treatment cost (per month average) |  |
| Out-of-pocket (RON) - amount spent | (numeric, integer) |
| Voluntary private insurance (RON) - amount spent | (numeric, integer) |
| Other costs with treatment (RON) - please specify what: | (open ended) |
| Other costs with treatment (RON) - amount spent | (numeric, integer) |

### Access To Medical Services

| Item | Response options |
| --- | --- |
| Please describe how often you usually access medical services for: |  |
| ATOPIC DERMATITIS (list several if applicable) |  |
| Types of visits to medical services in the last 12 months (multiple choice) (platform will generate clusters of questions based on number of services used) | Primary care (related to disease); Specialist physician (related to disease); Day hospitalization (related to disease); Inpatient hospitalization (related to disease); Laboratory; Imaging; Other (related to disease) |
| Number of services related to disease (last 12 months) | (numeric, integer) |
| Average visit duration, days (if applicable) | (numeric, integer) |
| Estimated service cost (per visit average) | (numeric, integer) |
| Out-of-pocket (RON) - amount spent | (numeric, integer) |
| Voluntary private insurance (RON) - amount spent | (numeric, integer) |
| Other costs with medical services (RON) - please specify what: | (open ended) |
| Other costs with medical services (RON) - amount spent | (numeric, integer) |
| ALOPECIA AREATA (list several if applicable) |  |
| Visits to medical services in the last 12 months (multiple choice) (platform will generate clusters of questions based on number of services used) | Primary care (related to disease); Specialist physician (related to disease); Day hospitalization (related to disease); Inpatient hospitalization (related to disease); Laboratory; Imaging; Other (related to disease) |
| Number of visits related to disease (last 12 months) | (numeric, integer) |
| Average visit duration (if applicable) | (numeric, integer) |
| Estimated service cost (per visit average) | (numeric, integer) |
| Out-of-pocket (RON) - amount spent | (numeric, integer) |
| Voluntary private insurance (RON) - amount spent | (numeric, integer) |
| Other costs with medical services (RON) - please specify what: | (open ended) |
| Other costs with medical services (RON) - amount spent | (numeric, integer) |

### End Of Questionnaire

| Item | Response options |
| --- | --- |
| Thank you for participating in this study. |  |

## Romanian version (versiunea în limba română)

### Eligibilitate

| Item | Response options |
| --- | --- |
| Introducere si consimtamant informat pentru evaluarea eligibilitatii | (din sablon atasat) - De acord/Nu sunt de acord |
| Aveti 18 ani impliniti? | Da/Nu |
| Sufar de (alegere multipla) | Dermatita atopica; Alopecia areata; Nici una dintre variante (redirectionare spre finalul chestionarului) |

### Introducere

| Item | Response options |
| --- | --- |
| Introducere si consimtamant informat | (din sablon atasat) - De acord/Nu sunt de acord |

### Demografice

| Item | Response options |
| --- | --- |
| Va rugam sa alegeti varianta care descrie cel mai bine situatia dumneavoastra: |  |
| Gen (alegere unica) | Femeie; Barbat; Alta varianta |
| Varsta | (numeric, intreg) |
| Mediul de resedinta (alegere unica) | Urban; Rural |
| Nivel de educatie (alegere unica) | Fara educatie formala; Scoala primara; Gimnaziu; Liceu; Scoala postliceala; Universitate |
| Statut angajare (alegere unica) | Student; Angajat norma intreaga; Angajat norma partiala; Somer; Pensionat; Alta varianta |

### Informatii Medicale

| Item | Response options |
| --- | --- |
| Va rugam sa alegeti varianta care descrie cel mai bine situatia dumneavoastra: |  |
| NHIH insurance status (alegere unica) | Asigurat; Neasigurat; Coasigurat; Nu stiu |
| Diagnostic medical de dermatita atopica (daca este cazul) | Am primit un diagnostic medical; Nu am primit un diagnostic medical |
| Diagnostic medical de alopecia areata (daca este cazul) | Am primit un diagnostic medical; Nu am primit un diagnostic medical |
| Luni de la diagnosticul de dermatita atopica (daca este cazul) | (numeric, intreg) |
| Luni de la diagnosticul de alopecia areata (daca este cazul) | (numeric, intreg) |
| Severitatea dermatitei atopice la diagnostic (daca este cazul) (alegere unica) | Usoara; Medie; Severa; Nu stiu |
| Controlul simptomelor dermatitei atopice in prezent (scala Likert cu patru optiuni) | Deloc sub control; Uneori sub control; Deseori sub control; Tot timpul sub control |
| Severitatea alopeciei areata la diagnostic (daca este cazul) (alegere unica) | S0 (0% cadere par); S1 (1-24% cadere par); S2 (25-49% cadere par); S3 (50-74% cadere par); S4 (75-99% cadere par); S5 (100% cadere par); Nu stiu |

### Utilizarea Tratamentului

| Item | Response options |
| --- | --- |
| Va rugam sa descrieti regimul actual de tratament: |  |
| DERMATITA ATOPICA (descrieti mai multe tratamente daca este cazul) |  |
| Numarul tratamentelor utilizare (platforma va genera seturi de intrebari pentru numarul indicat de respondent) | (numeric, intreg) |
| Numele tratamentului | (intrebare deschisa) |
| Tipul tratamentului (alegere unica) | Topic; Oral; Injectabil; Alta varianta |
| Doza | (intrebare deschisa) |
| Frecventa utilizarii (alegere unica) | O data pe saptamana; O data pe zi; De mai multe ori pe zi; La nevoie, in timpul puseelor; Nu stiu |
| Locatia administrarii | Acasa; La cabinetul medicului; La spital; Alta varianta |
| Cheltuieli estimate cu tratamentul (medie lunara) |  |
| Din buzunarul propriu (RON) - suma cheltuita | (numeric, intreg) |
| Asigurari private voluntare (RON) - suma cheltuita | (numeric, intreg) |
| Alte cheltuieli cu tratamentul (RON) - va rugam specificati tipul: | (intrebare deschisa) |
| Alte cheltuieli cu tratamentul (RON) - amount spent | (numeric, intreg) |
| ALOPECIA AREATA (if applicable) |  |
| Numarul tratamentelor utilizare (platforma va genera seturi de intrebari pentru numarul indicat de respondent) | (numeric, intreg) |
| Numele tratamentului | (intrebare deschisa) |
| Tipul tratamentului (alegere unica) | Topic; Oral; Injectabil; Alta varianta |
| Doza | (intrebare deschisa) |
| Frecventa utilizarii (alegere unica) | O data pe saptamana; O data pe zi; De mai multe ori pe zi; La nevoie, in timpul puseelor; Nu stiu |
| Locatia administrarii | Acasa; La cabinetul medicului; La spital; Alta varianta |
| Cheltuieli estimate cu tratamentul (medie lunara) |  |
| Din buzunarul propriu (RON) - suma cheltuita | (numeric, intreg) |
| Asigurari private voluntare (RON) - suma cheltuita | (numeric, intreg) |
| Alte cheltuieli cu tratamentul (RON) - va rugam specificati tipul: | (intrebare deschisa) |
| Alte cheltuieli cu tratamentul (RON) - amount spent | (numeric, intreg) |

### Acces La Servcii Medicale

| Item | Response options |
| --- | --- |
| Va rugam sa descrieti frecventa cu care utilizati servicii medicale pentru: |  |
| DERMATITA ATOPICA (descrieti mai multe servicii daca este cazul) |  |
| Tipul vizitelor la medic in ultimele 12 luni (alegere multipla) (platforma va genera seturi de intrebari pentru numarul indicat de respondent) | Medic de familie (in legatura cu boala); Medic specialist (in legatura cu boala); Spitalizare de zi (in legatura cu boala); Spitalizare continua/internare (in legatura cu boala); Laborator; Imagistica; Alta varianta (in legatura cu boala) |
| Numarul serviciilor accesate (in legatura cu boala) | (numeric, intreg) |
| Durata medie a vizitei, zile (daca este cazul) | (numeric, intreg) |
| Cheltuieli estimate cu serviciul medical (per visit average) | (numeric, intreg) |
| Din buzunarul propriu (RON) - suma cheltuita | (numeric, intreg) |
| Asigurari private voluntare (RON) - suma cheltuita | (numeric, intreg) |
| Alte cheltuieli cu servicii medicale (RON) - va rugam specificati tipul: | (intrebare deschisa) |
| Alte cheltuieli cu servicii medicale (RON) - amount spent | (numeric, intreg) |
| ALOPECIA AREATA (descrieti mai multe servicii daca este cazul) |  |
| Tipul vizitelor la medic in ultimele 12 luni (alegere multipla) (platforma va genera seturi de intrebari pentru numarul indicat de respondent) | Medic de familie (in legatura cu boala); Medic specialist (in legatura cu boala); Spitalizare de zi (in legatura cu boala); Spitalizare continua/internare (in legatura cu boala); Laborator; Imagistica; Alta varianta (in legatura cu boala) |
| Numarul serviciilor accesate (in legatura cu boala) | (numeric, intreg) |
| Durata medie a vizitei, zile (daca este cazul) | (numeric, intreg) |
| Cheltuieli estimate cu serviciul medical (per visit average) | (numeric, intreg) |
| Din buzunarul propriu (RON) - suma cheltuita | (numeric, intreg) |
| Asigurari private voluntare (RON) - suma cheltuita | (numeric, intreg) |
| Alte cheltuieli cu servicii medicale (RON) - va rugam specificati tipul: | (intrebare deschisa) |
| Alte cheltuieli cu servicii medicale (RON) - amount spent | (numeric, intreg) |

### Finalul Chestionarului

| Item | Response options |
| --- | --- |
| Va multumim pentru participarea in cadrul acestui studiu. |  |
